# Closed-Eye Visual Hallucinations Preceding Severe Alcohol Withdrawal

**DOI:** 10.7759/cureus.17040

**Published:** 2021-08-09

**Authors:** Saral Desai, Anca E Toma, Artem Sunik

**Affiliations:** 1 Department of Psychiatry, Brookdale University Hospital Medical Center, Brooklyn, USA; 2 Department of Neurology, Brookdale University Hospital Medical Center, Brooklyn, USA

**Keywords:** visual hallucinations, closed-eye visual hallucinations, alcohol withdrawal, psychiatry, neurology, hallucinations, substance use, delirium tremens, charles bonnet syndrome

## Abstract

Few existing cases of closed-eye visual hallucinations have been reported. These rare perceptual disturbances are distinct from open-eye visual hallucinations, as observed in Charles Bonnet syndrome. This case report discusses a 35-year-old male who presented with closed-eye visual hallucinations 24 hours before severe alcohol withdrawal. On initial presentation, the patient denied auditory or visual hallucinations. The day before the onset of severe alcohol withdrawal, the patient reported vivid, colorful, and lifelike visual hallucinations with his eyes closed which disappeared with eyes open. The hallucinations consisted of cartoon characters such as Daffy Duck, a lifelike blond woman with curly hair, and a tree-covered landscape. The patient endorsed awareness that the hallucinations were not real. The next day, the patient became acutely delirious with rapid onset of agitation, space-time disorientation, open-eye visual hallucinations, and emotionally disturbing illusions. The patient was promptly transferred to the intensive care unit (ICU) to manage severe alcohol withdrawal, including administration of intravenous sedation. During follow-up evaluation, the patient retained a clear memory of the closed-eye hallucinations. However, he had no recollection of the ICU course. Several etiologies were considered, including alcohol withdrawal complicated by polysubstance withdrawal and acute hepatitis, which resolved following pharmacological treatment. To the best of our knowledge, this is the first case report of its kind. Additional research is required to elucidate the etiology, mechanism, and clinical implications of closed-eye visual hallucinations.

## Introduction

Visual hallucinations are common in neurological and psychiatric disorders, such as schizophrenia, bipolar disorder with psychotic features, schizoaffective disorder, delirium, dementia, temporal lobe epilepsy, migraines, sleep disorders, substance use disorders, and neoplastic disorders [1]. Charles Bonnet syndrome is a known condition in which visual hallucinations occur due to visual sensory deprivation caused by vision loss and is most prevalent in elderly patients [2]. Previously published case reports describe a novel type of visual hallucination occurring primarily with eye closure and are distinct from the visual hallucinations associated with Charles Bonnet syndrome [3-8]. We present a case of closed-eye visual hallucinations with onset before severe alcohol withdrawal. Clinical significance, etiology, and treatment of such closed-eye visual hallucinations remain unknown.

## Case presentation

A 35-year-old male with prior history of major depressive disorder, traumatic brain injury, and severe polysubstance use (alcohol, cocaine, cannabis, opiates, and benzodiazepines) presented to the emergency department via emergency medical services after attempting to cut his wrists bilaterally in the setting of polysubstance use. In the emergency department, labs were remarkable for a blood alcohol level of 421 mg/dL and mild transaminitis (aspartate aminotransferase 325 U/L, alanine transaminase 284 U/L). The urine toxicology screen was positive for cannabinoids and benzodiazepines. On initial psychiatric evaluation, the patient was cooperative, tearful, and withdrawn. He endorsed suicidal ideation with depressed mood, anhedonia, hopelessness, and difficulty sleeping. During this time, the patient was alert and oriented to time, place, and person. He denied hallucinations, delusions, or other perceptual disturbances, and no psychosis was observed. The patient was medically cleared and transferred to the inpatient psychiatric unit for further stabilization. Within 72 hours, the patient was found to have worsening transaminitis (>1,500 U/L) and subsequently transferred to the regular medical floor to manage acute hepatitis. At this time, the ammonia level was normal (9 µmol/L), and mentation was intact.

The following day, the patient remained alert and oriented. He was cooperative, friendly, and maintained fair eye contact. His mood was neutral, his thought process logical and linear, and he denied suicidal or homicidal ideation. Physical examination was remarkable for mild bilateral hand tremors. The patient described new-onset closed-eye visual hallucinations, which resolved upon opening his eyes and recurred on eye closure. He denied auditory hallucinations, delusions, or other perceptual disturbance, and none were elicited. On further interrogation, the patient stated the hallucinations were vivid, graphic, and animated. He specifically reported observing the cartoon character Daffy Duck when he closed his eyes, which subsequently disappeared when he opened his eyes.

The next morning, the patient became disoriented, agitated, and presented with new-onset illusions, mistaking the treating psychiatrist for his father and the hospital bed for his living room couch. The patient was in severe emotional distress, asking the treatment team to help him save his father, whom he believed was trapped under the couch. The patient became increasingly agitated, attempted to elope by hitting his hospital room window with an IV pole, and was imminently transferred to the intensive care unit (ICU) to treat severe delirium, primarily attributed to alcohol withdrawal in the setting of suspected polysubstance withdrawal. At this time, the ammonia level was found to be elevated at 82 µmol/L. The patient was intubated for airway protection and started on intravenous (IV) midazolam 20 mg/hour and fentanyl 2,000 µg/hour.

After 72 hours, the patient was extubated, regained alertness and orientation, and followed complex commands. At this time, the patient denied hallucinations, delusions, illusions, or other perceptual disturbances. To further explore the nature of this unique clinical presentation, we gauged the patient’s level of insight regarding the hallucinations during a follow-up evaluation. The patient precisely recalled vivid closed-eye visual hallucinations. He confirmed the absence of visual hallucinations on admission. He also confirmed that the hallucinations began the day before severe delirium, though he stated he had little to no recollection of his ICU course. The patient also confirmed that the hallucinations appeared when he closed his eyes and resolved when he opened his eyes. Each time he closed his eyes, the patient recalled watching a different scene or subject, occurring in nonsequential order, without distinct features. The patient described seeing cartoon characters such as Daffy Duck, an unfamiliar blond woman with curly hair having an ambiguous face, and a forested landscape. He described the images as vibrant, lifelike, and evocative. He denied the images were memories or personally relevant and stated he knew these visual hallucinations were not real. Although the patient admitted to a prior history of delirium tremens, alcohol-related blackouts, polysubstance use, and polysubstance withdrawal, the patient stated the closed-eye visual hallucinations occurred for the first time.

## Discussion

Visual hallucinations are frequently observed in various neurological conditions, including Lewy body dementia, temporal lobe epilepsy, and Parkinson’s disease [1]. Additionally, they are observed in psychiatric conditions such as psychosis and substance intoxication or substance withdrawal [1]. Delirium is another condition frequently associated with visual hallucinations [1]. Additionally, hypnagogic and hypnopompic visual hallucinations commonly occur during transitions of wakefulness to sleep and vice versa, respectively [9]. Finally, visual hallucinations may occur in narcolepsy during the transition from rapid eye movement into wakefulness [9].

Closed-eye visual hallucinations are similar to those seen in Charles Bonnet syndrome in that both occur in the absence of visual sensory input [2]. The visual hallucinations described by our patient were silent, noninteractive, and impersonal, much like the ones described in Charles Bonnet syndrome, and differing from the hallucinations characteristic of psychosis [10].

The visual hallucinations described by our patient also differ from those seen in Charles Bonnet syndrome in several ways. First, our patient’s vision is intact. Second, he only experienced visual hallucinations upon eye closure. This contrasts with Charles Bonnet syndrome, characterized by visual hallucinations with open eyes that resolve upon eye closure [10]. We consider the possibility that our patient’s intact afferent visual input may be dominant when his eyes are open, which may override an internally generated signal in the visual cortex responsible for the visual hallucinations. Charles Bonnet syndrome is characterized by visual impairment resulting in sensory deafferentation, leading to disinhibition of the visual cortex leading to visual hallucinations [11]. Our patient’s closed-eye visual hallucinations may have occurred from disinhibition of the visual cortex in the absence of visual input on the eye closure due to an imbalance between excitatory and inhibitory neurotransmitters due to alcohol and polysubstance withdrawal. However, further research is required to determine the exact mechanism and its clinical implications.

At times mistaken for visual hallucinations, phosphenes are spontaneous and luminous intrinsic visual phenomena present as compound geometric shapes [12,13]. In contrast to the content of visual hallucinations, luminous phenomena such as phosphenes or white noise are ambiguous shapes unrelated to cultural references [12,13]. This distinction is strengthened when the visual hallucinations are associated with psychoactive substance use, mood disturbance, or in the setting of acute conditions such as fever or psychosis [12,13]. Although it cannot be ruled out, we believe our patient’s sensory experience is unlikely attributed to phosphenes.

A literature search revealed a limited number of published case reports of closed-eye visual hallucinations at the time of submission, summarized in Table 1. The majority of cases describe patients with closed-eye visual hallucinations in the absence of delirium. Most patients acknowledge that the hallucinations were not real. Most patients describe the hallucinations as vivid, lifelike images, resolving with either correction of underlying etiology, or more commonly, spontaneously without treatment. Reported symptom duration ranges between four hours to eleven days.

**Table 1:** Published case reports describing closed-eye visual hallucinations CABG: coronary artery bypass graft

Year	Author	Type of hallucination	Duration	Suspected etiology	Treatment
2018	Peck et al. [3]	A lifelike colorful moving car driving in front of the patient	Six days	Hyponatremia	Resolved after correction of hyponatremia
2011	Weinschenk et al. [4]	Vivid and realistic images of glaciers, waterfalls, and other picturesque landscapes	Three days	Lidocaine use	Spontaneous resolution
2008	Otomo et al. [5]	Vivid, realistic images of colored clothes, lace curtains, handbags, and sofas	Four days	Postoperative after epidural with general anesthesia	Spontaneous resolution
2005	Eissa et al. [6]	Vivid images of pipes, tubing, toolmaking machinery, and front view of a church	Three days	Neurologic insult during CABG	Spontaneous resolution
1991	Fisher et al. [7]	Colorful, lifelike images of individual letters, landscapes, and football players	Eleven days	Atropine toxicity	Spontaneous resolution
1991	Fisher et al. [8]	Colorful images of books, snowy landscapes, and various animals	Four hours	Lidocaine use	Spontaneous resolution

Our patient experienced closed-eye visual hallucinations preceding severe alcohol withdrawal. While visual hallucinations are known during alcohol withdrawal, prodromal closed-eye visual hallucinations such as those described in our case have not been reported [14]. The patient also denied any prior occurrence of closed-eye visual hallucinations during polysubstance use or withdrawal. Finally, we considered that the closed-eye visual hallucinations might have occurred due to hepatic encephalopathy. However, the patient’s ammonia levels were normal when he reported closed-eye visual hallucinations, despite moderate-to-severe transaminitis. His blood ammonia levels became abnormal the following day, coinciding with the onset of severe delirium attributed to acute hepatitis. Finally, given the patient’s initial suicidality and eventual severe agitation, we considered whether the visual hallucinations might have occurred in the setting of psychosis. However, the patient’s hallucinations were impersonal, unrelated to his thoughts or feelings, and did not speak to him directly. This contrasts with psychotic hallucinations, which are usually either ego-dystonic or ego-syntonic and interactive [15]. Furthermore, visual hallucinations attributed to psychosis are often accompanied by other features of psychosis such as delusions, disorganized thought processes, and behavior [15]. None of these were reported or observed when the patient was experiencing closed-eye visual hallucinations.

The closed-eye visual hallucinations reported by our patient are similar to the ones previously described in a few limited cases, distinct from the visual hallucinations described in Charles Bonnet syndrome or psychotic disorders [3-8]. Additional research is required to elucidate the mechanism, etiologies, and clinical implications of this curious phenomenon.

## Conclusions

In contrast to the less complex and geometric visual hallucinations associated with visual impairment described in Charles Bonnet syndrome, or the interactive and disturbing visual hallucinations of psychosis, closed-eye visual hallucinations are vivid, animated yet impersonal, and are not always associated with delirium. As evidenced in this case study, closed-eye visual hallucinations may precede severe delirium. In the setting of alcohol withdrawal, the perceptual disturbance may resolve with treatment of underlying pathology. However, further research is necessary to elucidate the mechanism, specific etiology, and clinical implications of this intriguing phenomenon for prompt diagnosis and timely administration of life-saving treatment.
